# Every second counts: the association of embryo transfer duration with live birth following 2267 single, euploid, frozen embryo transfer

**DOI:** 10.1007/s10815-025-03520-7

**Published:** 2025-06-05

**Authors:** Blake Vessa, Devika Sachdev, Leah Roberts, Kara Scarpetti, Christine Whitehead, Maria Costantini, Paul Bergh, Marie Werner, Kassie Bollig

**Affiliations:** 1 IVIRMA Global Research Alliance, IVIRMA New Jersey, 140 Allen Road, Basking Ridge, NJ 07920 USA; 2 Sidney Kimmel Medical College, Thomas Jefferson, Philadelphia, PA USA; 3 Boca Fertility IVF Clinic Center, Boca Raton, FL USA; 4 Department of Obstetrics, Gynecology & Reproductive Sciences, Rutgers Robert Wood Johnson Medical School, New Brunswick, NJ USA

**Keywords:** Embryo transfer duration, IVF outcomes, Pregnancy, Live birth

## Abstract

**Purpose:**

To identify the impact of the duration of the embryo transfer procedure on pregnancy outcomes.

**Methods:**

This was a retrospective cohort study at a single center including single euploid frozen embryo transfers from April to December 2022. Embryo transfer time was defined as the total time (seconds) from when the embryologist handed the catheter with the loaded embryo to the physician, to the release of the embryo into the uterus. Time measurements were divided into quartiles: (1) 4–27 s, (2) 28–38 s, (3) 39–54 s, and (4) 55–1028 s. The primary outcome was the probability of live birth per transfer. Secondary outcomes included biochemical pregnancy, clinical pregnancy, and pregnancy loss.

**Results:**

A total of 2267 frozen embryo transfer cycles were included in the analysis. The overall live birth rate was 59.8% (1356/2267). Compared to the fastest time (1st quartile), the probability of live birth was significantly decreased for couples with the longest transfer times (4th quartile) after adjusting for possible confounding variables (aOR, 0.76; 95% CI 0.58–0.99; *p* = 0.046). When patients with more difficult embryo transfers, that as a result may be inherently longer, were excluded, the results were the same as the primary model. However, when data was analyzed using only first transfers, compared to the shortest transfer time quartile (1st), there was a significant decreased probability of live birth in the 3rd quartile (OR 0.63, 95% CI 0.41–0.94, *p* = 0.025), but no significant differences in the 2nd or 4th quartile groups (*p* = 0.213, *p* = 0.800). There were no differences in the probability of biochemical pregnancy, clinical pregnancy, or pregnancy loss in the fully adjusted model.

**Conclusion:**

The longest embryo transfer times are associated with significantly decreased probability of live birth.

**Supplementary Information:**

The online version contains supplementary material available at 10.1007/s10815-025-03520-7.

## Introduction

The embryo transfer (ET) is the final and most essential step in the in vitro fertilization (IVF) process. As a result, many studies have focused on modifiable techniques to improve transfer success rates. There is reproducible data to support several techniques that have been widely agreed upon and are included in the embryo transfer guidelines from the Practice Committee of the American Society of Reproductive Medicine, including the use of abdominal ultrasound and a soft catheter, removal of cervical mucus, placement of the catheter tip in the upper or middle area of the uterine cavity, and immediate ambulation after the ET to improve pregnancy rates [1–8]. However, variations in patient characteristics, clinical practices, skill of providers, and technology across clinics make it challenging to standardize techniques and gather consistent, generalizable data [9]. Specifically, determining if there is an ideal length of time for the ET procedure to be performed within, or more precisely the time where the embryo is no longer in the controlled environment of the embryology culture media and before it is successfully expelled into the uterine cavity, has not been sufficiently studied. As a result, neither the guidelines from the Practice Committee of the American Society of Reproductive Medicine nor recent systematic reviews and meta-analyses specify any cutoffs for transfer time [1, 8].

The ET process, while typically brief, involves delicate handling of the embryo with its precise placement into the uterine cavity. There is limited data suggesting that longer ET times may be associated with lower pregnancy success rates, whereas a swift and smooth ET may reduce these risks, potentially improving outcomes [10]. This may be due to increased exposure of the embryo to suboptimal conditions, mechanical stress during the transfer, or heightened uterine contractions. However, the two studies showing an association between longer transfer times and lower pregnancy rates were both comprised of only fresh IVF transfers [10, 11], which may not be generalizable to today’s practice. The first study published in 2004 including 450 fresh ETs concluded transfers resulting in a pregnancy were on average 10 s shorter compared to unsuccessful ETs [11]. A similar 2010 study that included a combination of day 3 and day 5 fresh transfers concluded there were significantly lower pregnancy rates in transfers that exceeded 60 s in duration [10].

On the contrary, a 2015 prospective cohort study of 410 fresh IVF transfers found that the duration of the ET did not have a significant effect on either ongoing pregnancy or live birth [12]. Most recently, Lee et al. [13] examined this relationship in a retrospective cohort study of 465 blastocyst FETs utilizing a hormone replacement (programmed) protocol from a single center between 2007 and 2014 and found longer ET times did not negatively affect clinical pregnancy rate (CPR), implantation rate (IR), or live birth rate (LBR) after adjusting for potential confounders. The restrictive population utilized that accounted for important confounders and optimized uterine receptivity by including only frozen transfers may have contributed to their contrasting conclusion [13]. However, this study still posed some major limitations to the application to modern practice: all embryos were cryopreserved using slow freezing, all cycles were programmed protocols, donor oocytes were included in analyses, some transfers were double embryo transfers (DETs), and they included both first and second FETs [13]. These outdated techniques, specifically slow freezing and DETs, are not generalizable to today’s standard practice, with advanced technology supporting vitrification and single euploid or high-quality embryo transfers (SETs). Additionally, the use of a SET protocol limits generalizability to the broader infertility population. Although appropriate statistical modeling was used to analyze multiple cycles from the same patient, residual bias may exist from physician knowledge of a prior embryo transfer outcome.

Therefore, the existing data is limited and contradictory, but most importantly, not reflective of current clinical practice techniques and standards. As a result, using this older data may be potentially misleading. There are no published studies including only single, euploid, FETs with embryos cryopreserved by vitrification, the current standard of care. Therefore, the objective of this study is to identify the impact the duration of the ET has on pregnancy outcomes while accounting for the limitations of prior investigations. Understanding the relationship between ET duration and pregnancy success rates may offer valuable insights into improving clinical practices and patient outcomes for those undergoing IVF.

## Materials and methods

### Study population

This was a retrospective cohort study completed at a single academic-affiliated center investigating the association between embryo transfer time and live birth. The study population included single euploid frozen embryo transfers from April 2022 to December 2022 using autologous oocytes and sperm. All study participants underwent a vaginal oocyte retrieval at a single IVF center. The study did include patients who may have had prior embryo transfers before April 2022. Prior to proceeding with FET, all patients also had a uterine cavity evaluation with a hysterosalpingogram or saline-infusion sonogram in addition to a mock ET catheter check as is standard protocol at our institution. At the time of ET, the direct transfer technique was utilized [14]. Patients using surgically extracted sperm, oocytes or embryos that had originated from an outside laboratory, gestational carriers, or thawed and re-vitrified and/or trophectoderm re-biopsied embryos were excluded. Institutional review board approval was obtained to evaluate the retrospective data (Advarra IRB, Pro00027158).

### Exposure and primary outcome

Embryo transfer time was defined as the total time (seconds) from when the embryologist first handed the catheter with the loaded embryo to the physician to the final release of the embryo into the uterine cavity, confirmed with transabdominal ultrasound guidance. There was a single clinical assistant recording this time for all of the patients included in this study. To examine if a specific cutoff point exists where time may impact ET outcomes, time measurements were divided into the following quartiles: (1) 4–27 s, (2) 28–38 s, (3) 39–54 s, and (4) 55–1028 s. The primary outcome was the probability of live birth per ET. Secondary outcomes included biochemical pregnancy (serum beta-hcg > 5 mIU), clinical pregnancy (defined as presence of intrauterine gestational sac and yolk sac on ultrasound), and pregnancy loss (spontaneous miscarriage and/or stillbirth (loss prior to 22 weeks gestation)).

### Frozen embryo transfer and embryology protocols

Three different FET protocols are utilized at our institution and are determined based on individual patient factors and physician preference as follows: (1) natural, (2) stimulated, and (3) programmed FET cycles. Prior to initiation of all cycles, a baseline assessment is conducted between days 2–4 of the menstrual cycle, which includes a pelvic ultrasound and serum measurements of estradiol (E2), progesterone (P4), and beta human chorionic gonadotropin (hCG). For natural FET cycles, repeat ultrasound and blood work (E2, P4, and luteinizing hormone (LH)) is typically performed on day 10 and then repeated every 1–3 days until a dominant follicle is present, E2 is > 150–200 pg/mL, and endometrial thickness (EMT) is >/= 7 mm with a type 1 (trilaminar) pattern. The dominant follicle is either triggered with recombinant hCG when it reaches a mean sac diameter (MSD) > 18 mm or when a natural LH surge occurs. Vaginal Prometrium (200 mg twice daily) is then routinely started 12–36 h after the natural LH surge or the hCG trigger. For stimulated cycles, controlled ovarian stimulation is accomplished with either Letrozole, Clomiphene Citrate, or injectable gonadotropins, with varying doses selected based on patient characteristics and provider preference. Similar to natural cycles, once a dominant follicle reaches a MSD > 18 mm and EMT is ≥ 7 mm, then a recombinant hCG trigger is used and vaginal Prometrium is initiated 48 h later. For programmed cycles, typically oral estrace is initiated at a dose of 2 mg BID and increased to TID after 4 to 5 days. Alternative dosing protocols include vaginal estrace utilized at a dose of 2 mg BID and/or estrogen patches, depending on individual provider preference. Monitoring involves regular ultrasounds and blood work to assess the growth of the endometrial lining and hormone levels. Once the EMT is ≥ 7 mm, progesterone in oil (PIO) is initiated at a dose of 50 mg daily. This dose may be adjusted based on serial blood work measurements, with the ideal serum P4 level maintained ≥ 12 ng/mL. The ET is scheduled based on the day of progesterone initiation: approximately 120 h of progesterone exposure or 5 days after progesterone initiation.

The embryology laboratory utilizes a modified Gardner blastocyst grading system in addition to standardized protocols for embryo vitrification and thawing. All embryos transferred in this study population underwent pre-implantation genetic testing for aneuploidy (PGT-A) and were euploid. The vitrification protocol utilized in the laboratory consists of a two-step process. First, embryos for cryopreservation are placed into an equilibrating solution (ES) consisting of 7.5% DMSO + 7.5% EG for 12–15 min. The second step is a short (30–90 s) exposure to the vitrification solution (VS) containing higher cryoprotective additives (15% DMSO + 15% EG) as well as a dehydrating agent (sucrose). This is carried out at room temperature. All cryopreserved embryos are then stored under liquid nitrogen at − 196 °C. The thawing process is also a standardized protocol consisting of a slow three-step dilution of cryoprotective additives and sucrose, with the first step performed at 37 °C and the subsequent steps performed at room temperature.

At the time of the ET procedure, the embryologist brings the embryo into the room in a temperature and pH-controlled isolette. All of the ET rooms are small 117 square foot rooms, so that the embryologist does not need to walk with the embryo in the catheter, but instead is able to hand the catheter to the physician performing the ET directly from the isolette when ready.

### Statistical analysis

Univariate descriptive analyses for baseline demographic and clinical characteristics were analyzed using ANOVA and Kruskal–Wallis for continuous variables, and Pearson’s chi-square test or Fisher’s exact test for categorical variables. Multivariate logistic regression was used to examine the relationship between embryo transfer time and the primary outcome of live birth. Potential confounders were selected a priori based on biologic plausibility and previous supporting literature [1, 13]. Covariates selected for inclusion in the final model included oocyte age (year), maternal body mass index (BMI, kg/m^2^), primary infertility diagnosis, FET protocol (natural, stimulated, programmed), EMT (mm) prior to ovulation or exogenous progesterone start, maximum E2 level (pg/mL) prior to ovulation or exogenous progesterone start, embryo grade (Gardner grading system: day of blastulation, expansion, inner cell mass (ICM), and trophectoderm grade), uterine position (anteverted or retroverted), presence of flash at time of ET (air bubble visually seen on transabdominal ultrasound at the time of expulsion from the transfer catheter; yes/no), embryo thaw time (minutes from thaw to embryo expulsion in the uterine cavity), history of prior embryo transfer (yes/no), physician performing the transfer, and embryologist performing the transfer. Catheter type was not adjusted for because 94% of ETs utilized the Wallace 18 catheter. Additionally, the ET catheter being handed back to embryologist, stylet use, or physician labeling an ET as “difficult” were not controlled for in the logistic regression because these by default contribute to increased ET time, and inclusion of these covariates in the model would lead to over-adjustment for the main exposure potentially leading to an underestimation of the true effect. From the adjusted model, we also conducted a linear test for trend to assess if the probability of live birth systematically increased or decreased over the ordered levels of embryo transfer time quartiles after accounting for possible confounders.

To assess the robustness of the dataset, several sensitivity analyses were conducted. The first included using data from patients undergoing their first FET cycles only. This approach aimed to eliminate any potential confounding effects of a patient having a previous FET cycle, such as a physician “learning” from a prior transfer. Two restriction analyses were also performed examining factors inherently known to increase transfer time. The first excluded cases where (1) a stylet was used, (2) the embryo catheter was handed back to the embryologist, or (3) a transfer was deemed “difficult” by the performing physician. The second analysis excluded returned (embryo retained in the catheter after deployment) and reloaded (embryo expelled from one catheter and reloaded into another) embryos. Both analyses aimed to further isolate the direct impact of time on pregnancy outcomes and to avoid potential residual unmeasured confounding that may be present in these specific cases.

After accounting for all inclusion criteria, only 1.8% (42/2309) of the total cases were excluded due to missing information on BMI, endometrial morphology prior to ovulation trigger or administration of exogenous progesterone, or embryo thaw time. Ninety-eight percent of the cases had no missing covariates, and therefore complete case analysis was utilized with low probability of any selection bias. All analyses were carried out using STATA software version 17.0 (College Station, Texas).

## Results

A total of 2267 frozen embryo transfer cycles from 2267 unique patients were included in this analysis. A total of 18 physicians performed an average of 126 transfers each (range of 13–260 transfers per physician) (Supplemental Table 1A). A total of 13 embryologists performed an average of 174 transfers each (range 30–321 transfers per embryologist) (Supplemental Table 1B). The number of ETs per time quartile was as follows: (1) 4–27 s (*n* = 593), (2) 28–38 s (*n* = 577), (3) 39–54 s (*n* = 535), and (4) 55–1028 s (*n* = 562). The distribution of transfer times in each quartile is provided in Supplemental Fig. 1. Baseline cycle and patient characteristics are shown in Table 1 in addition to the Society of Assisted Reproductive Technology (SART) embryo grades. Longer ET times had a higher proportion of first FET cycles (*p* = 0.024). Women with anteverted uteri were more likely to have shorter ET times (88.5% in quartile 1 vs. 77.2% in quartile 4, *p* < 0.001) and shorter ET times were more likely to have the presence of a flash on ultrasound (*p* = 0.001).

**Table 1:** Baseline patient and cycle characteristics

	Total	1st quartile (4–27 s)	2nd quartile (28–38 s)	3rd quartile (39–54 s)	4th quartile (55–1028 s)	*p*-value
	*N* = 2267	*N* = 593	*N* = 577	*N* = 535	*N* = 562	
Oocyte age (years)	34.5 (4.1)	34.7 (3.9)	34.3 (4.1)	34.5 (4.1)	34.5 (4.4)	0.39
Maternal age at embryo transfer (years)	35.5 (4.2)	35.7 (4.0)	35.4 (4.2)	35.5 (4.1)	35.4 (4.5)	0.42
Body mass index (kg/m^2^) at time of embryo transfer	25.6 (22.3–30.6)	25.7 (22.5–29.9)	25.6 (22.5–30.7)	25.2 (22.3–30.1)	25.7 (22.2–31.3)	0.84
Body mass index (kg/m^2^) at time of embryo transfer (categorical)						0.28
Normal weight	999 (44.1%)	253 (42.7%)	251 (43.5%)	249 (46.5%)	246 (43.8%)	
Underweight	47 (2.1%)	11 (1.9%)	15 (2.6%)	11 (2.1%)	10 (1.8%)	
Overweight	610 (26.9%)	181 (30.5%)	154 (26.7%)	140 (26.2%)	135 (24.0%)	
Obese	611 (27.0%)	148 (25.0%)	157 (27.2%)	135 (25.2%)	171 (30.4%)	
Primary infertility diagnosis						0.68
Unexplained	166 (7.3%)	44 (7.4%)	33 (5.7%)	41 (7.7%)	48 (8.5%)	
Male factor	761 (33.6%)	198 (33.4%)	189 (32.8%)	189 (35.3%)	185 (32.9%)	
Genetic	109 (4.8%)	20 (3.4%)	33 (5.7%)	21 (3.9%)	35 (6.2%)	
Mullerian anomaly	6 (0.3%)	0 (0.0%)	2 (0.3%)	3 (0.6%)	1 (0.2%)	
Tubal factor	108 (4.8%)	30 (5.1%)	24 (4.2%)	26 (4.9%)	28 (5.0%)	
Endometriosis	78 (3.4%)	18 (3.0%)	24 (4.2%)	17 (3.2%)	19 (3.4%)	
Diminished ovarian reserve	352 (15.5%)	95 (16.0%)	95 (16.5%)	76 (14.2%)	86 (15.3%)	
Fertility preservation	15 (0.7%)	4 (0.7%)	2 (0.3%)	4 (0.7%)	5 (0.9%)	
Procreative management	34 (1.5%)	6 (1.0%)	6 (1.0%)	9 (1.7%)	13 (2.3%)	
Other uterine factor	52 (2.3%)	12 (2.0%)	12 (2.1%)	16 (3.0%)	12 (2.1%)	
Ovulatory dysfunction	449 (19.8%)	123 (20.7%)	122 (21.1%)	103 (19.3%)	101 (18.0%)	
Recurrent pregnancy loss	137 (6.0%)	43 (7.3%)	35 (6.1%)	30 (5.6%)	29 (5.2%)	
First FET cycle						0.024
Yes	1110 (49.0%)	286 (48.2%)	267 (46.3%)	251 (46.9%)	306 (54.4%)	
No	1157 (51.0%)	307 (51.8%)	310 (53.7%)	284 (53.1%)	256 (45.6%)	
ET protocol						0.25
Natural	433 (19.1%)	117 (19.7%)	125 (21.7%)	101 (18.9%)	90 (16.0%)	
Stimulated	118 (5.2%)	30 (5.1%)	34 (5.9%)	23 (4.3%)	31 (5.5%)	
Programmed	1716 (75.7%)	446 (75.2%)	418 (72.4%)	411 (76.8%)	441 (78.5%)	
Maximum endometrial thickness prior to ovulation or exogenous progesterone (mm)	8.9 (8.0–10.3)	9.0 (8.0–10.2)	8.9 (8.0–10.2)	8.9 (8.0–10.3)	9.0 (8.0–10.5)	0.82
Endometrial morphology prior to ovulation or exogenous progesterone						0.46
1	2175 (95.9%)	571 (96.3%)	549 (95.1%)	514 (96.1%)	541 (96.3%)	
2	77 (3.4%)	19 (3.2%)	26 (4.5%)	17 (3.2%)	15 (2.7%)	
3	15 (0.7%)	3 (0.5%)	2 (0.3%)	4 (0.7%)	6 (1.1%)	
Estradiol level prior to ovulation or exogenous progesterone (pg/mL)	233.0 (171.0–326.0)	244.0 (177.7–329.9)	221.0 (167.0–327.0)	236.6 (169.0–319.6)	230.0 (169.0–326.0)	0.22
Progesterone level prior to ovulation or exogenous progesterone (ng/mL)	0.4 (0.2–0.5)	0.4 (0.3–0.5)	0.4 (0.2–0.5)	0.4 (0.2–0.5)	0.4 (0.2–0.5)	0.70
Endometrial thickness on the day prior to embryo transfer (mm)	9.7 (8.2–11.5)	9.9 (8.3–11.3)	9.6 (8.2–11.5)	9.7 (8.0–11.6)	9.9 (8.3–11.7)	0.65
Endometrial morphology on the day prior to embryo transfer						0.91
1	11 (0.5%)	3 (0.5%)	2 (0.3%)	4 (0.7%)	2 (0.4%)	
2	91 (4.0%)	20 (3.4%)	24 (4.2%)	23 (4.3%)	24 (4.3%)	
3	2165 (95.5%)	570 (96.1%)	551 (95.5%)	508 (95.0%)	536 (95.4%)	
Estradiol level on day prior to embryo transfer (pg/mL)	158.0 (114.0–228.0)	151.0 (112.0–220.0)	152.0 (108.0–222.0)	164.0 (117.0–228.0)	162.0 (117.0–244.7)	0.05
Progesterone level on day prior to embryo transfer (ng/mL)	22.5 (17.0–29.4)	22.4 (17.7–29.0)	22.2 (17.3–28.4)	23.0 (16.4–30.6)	22.6 (16.9–30.0)	0.87
Total thaw time prior to ET (minutes)	301.0 (277.0–328.0)	300.0 (275.0–325.0)	300.0 (277.0–324.0)	301.0 (276.0–331.0)	303.0 (277.0–331.0)	
Day of blast						0.53
5	817 (36.0%)	222 (37.4%)	221 (38.3%)	184 (34.4%)	190 (33.8%)	
6	1372 (60.5%)	351 (59.2%)	341 (59.1%)	331 (61.9%)	349 (62.1%)	
7	78 (3.4%)	20 (3.4%)	15 (2.6%)	20 (3.7%)	23 (4.1%)	
Grade of embryo expansion						0.88
3	8 (0.4%)	3 (0.5%)	2 (0.3%)	1 (0.2%)	2 (0.4%)	
4	799 (35.2%)	210 (35.4%)	194 (33.6%)	200 (37.4%)	195 (34.7%)	
5	834 (36.8%)	215 (36.3%)	228 (39.5%)	190 (35.5%)	201 (35.8%)	
6	626 (27.6%)	165 (27.8%)	153 (26.5%)	144 (26.9%)	164 (29.2%)	
Grade of inner cell mass						0.074
A	692 (30.5%)	205 (34.6%)	175 (30.3%)	155 (29.0%)	157 (27.9%)	
B	1363 (60.1%)	342 (57.7%)	346 (60.0%)	318 (59.4%)	3457 (63.5%)	
C	212 (9.4%)	46 (7.8%)	56 (9.7%)	62 (11.6%)	48 (8.5%)	
Grade of trophectoderm						0.51
A	509 (22.5%)	149 (25.1%)	125 (21.7%)	118 (22.1%)	117 (20.8%)	
B	1456 (64.2%)	371 (62.6%)	379 (65.7%)	346 (64.7%)	360 (64.1%)	
C	302 (13.3%)	73 (12.3%)	73 (12.7%)	71 (13.3%)	85 (15.1%)	
SART embryo grades						0.069
Good	688 (30.3%)	205 (34.6%)	173 (30.0%)	155 (29.0%)	155 (27.6%)	
Fair	1363 (60.1%)	342 (57.7%)	346 (60.0%)	318 (59.4%)	357 (63.5%)	
Poor	216 (9.5%)	46 (7.8%)	58 (10.1%)	62 (11.6%)	50 (8.9%)	
Position of uterus						< 0.001
Anteverted	1896 (83.6%)	525 (88.5%)	492 (85.3%)	445 (83.2%)	434 (77.2%)	
Retroverted	371 (16.4%)	68 (11.5%)	85 (14.7%)	90 (16.8%)	128 (22.8%)	
Presence of flash on ultrasound						0.001
No	64 (2.8%)	9 (1.5%)	12 (2.1%)	14 (2.6%)	29 (5.2%)	
Yes	2203 (97.2%)	584 (98.5%)	565 (97.9%)	521 (97.4%)	533 (94.8%)	

Data are presented as mean (SD) or median (IQR) for continuous measures, and *n* (%) for categorical measures

*FET* frozen embryo transfer, *ET* embryo transfer, *SART* Society of Assisted Reproductive Technology

**Fig. 1:**
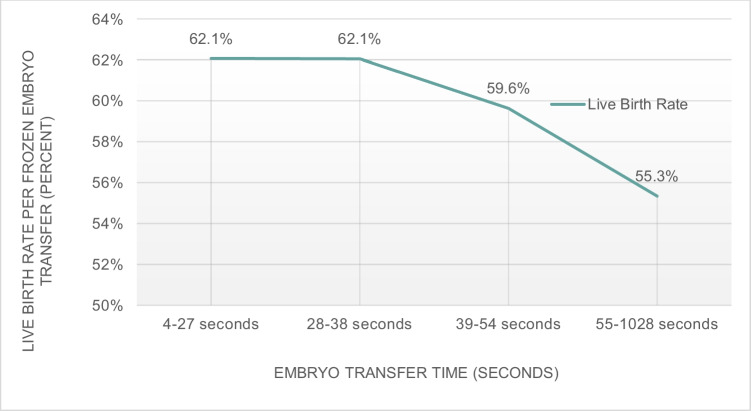
Live birth rate and embryo transfer time (quartiles). Live birth rate (percent) for each embryo transfer time quartile

The primary outcome was the probability of live birth. The overall live birth rate (LBR) was 59.8% (1356/2267). Figure 1 describes the LBR for each embryo transfer time quartile. The mean ET time that resulted in a live birth was 53.9 s compared to 59.9 s in cycles that did not result in a live birth (*p* = 0.071). Compared to the fastest time (1st quartile), the probability of live birth was significantly decreased for couples with the longest ET times (4th quartile) after adjusting for possible confounding variables (oocyte age, maternal BMI, infertility diagnosis, ET protocol, endometrial thickness, estradiol level, day of blastulation, embryo expansion grade, embryo inner cell mass grade, embryo trophectoderm grade, uterine position, presence of flash, embryo thaw time, history of prior transfer, physician, and embryologist) (aOR, 0.76; 95% CI 0.58–0.99; *p* = 0.046). No statistically significant differences were seen in the probability of live birth in the second or third ET time quartiles, when the first quartile (shortest transfer time) was the comparator group. In pairwise comparisons between other combinations of ET time quartiles (3rd quartile vs. 2nd quartile; 4th quartile vs. 2nd quartile; 4th quartile vs. 3rd quartile), there was also a significant decrease in the probability of live birth in fully adjusted models when comparing the 1st and 3rd quartiles (*p* = 0.046), but not between other quartile comparisons. However, there was a significant dose–response relationship between ET duration and probability of live birth in linear trend adjusted analyses (*p* = 0.014).

**Fig. 2:**
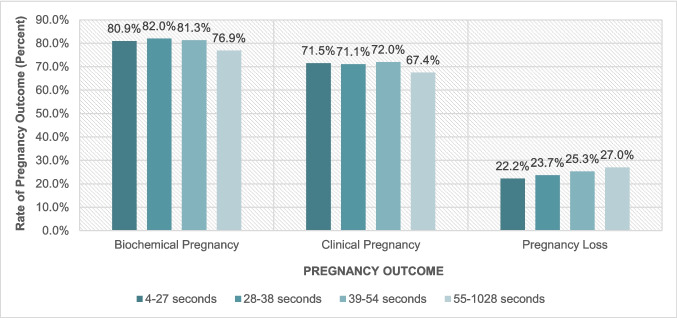
Biochemical, clinical, and pregnancy loss rates and embryo transfer time (quartiles). Biochemical pregnancy, clinical pregnancy and pregnancy loss rates (percent) for each quartile

**Fig. 3:**
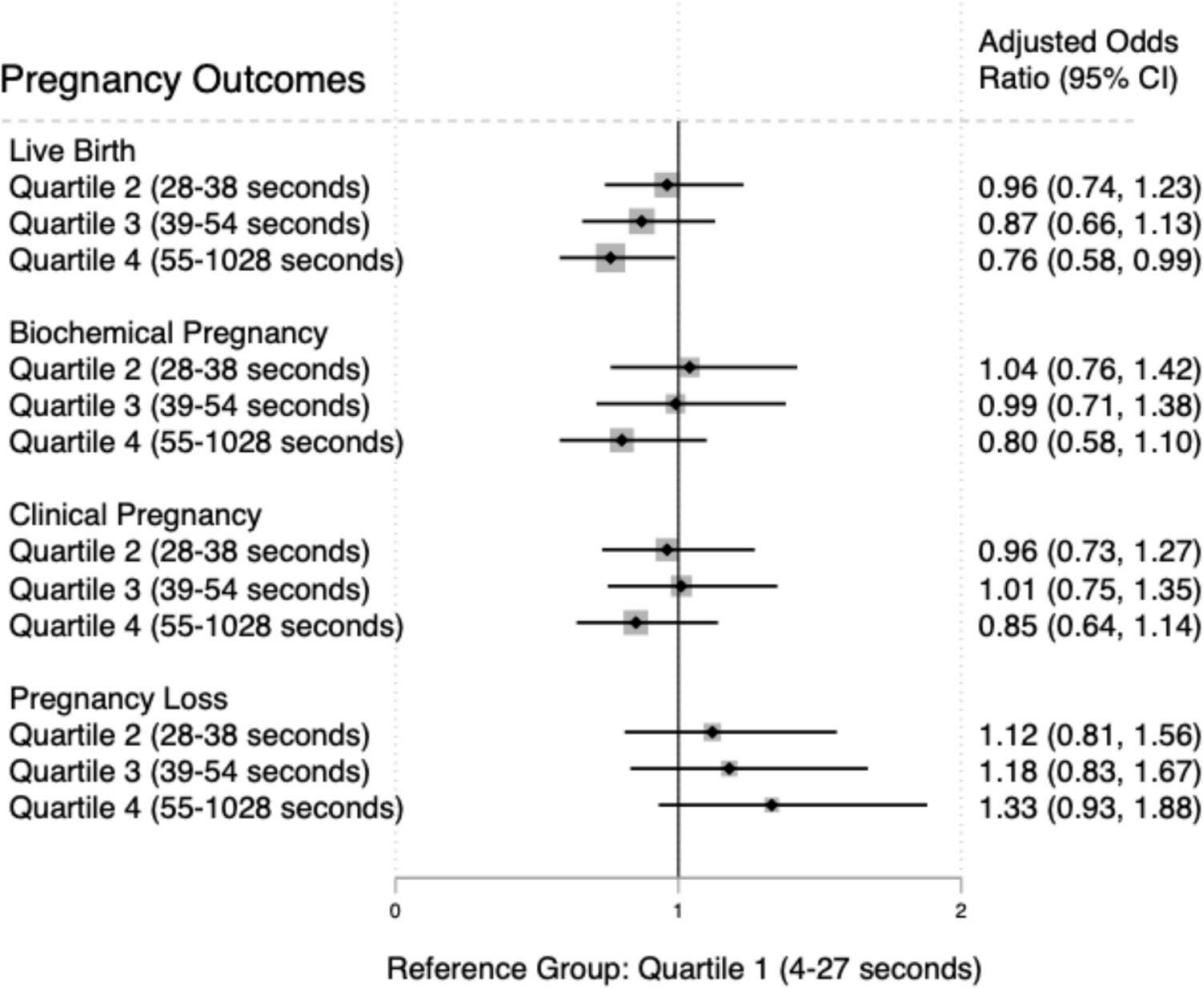
Association of embryo transfer time with pregnancy outcomes. Forest plot showing a significantly lower probability (odds) of live birth in the fully adjusted model between the 1st quartile (shortest embryo transfer times) and the 4th quartile (longest embryo transfer times). There were no differences in the probability (odds) of biochemical pregnancy, clinical pregnancy, or pregnancy loss in the fully adjusted model. Multivariate model was adjusted for oocyte age, maternal body mass index, primary infertility diagnosis, transfer protocol, endometrial thickness, estradiol level, day of blastulation, embryo expansion grade, inner cell mass grade, trophectoderm grade, uterine position, presence of flash, embryo thaw time, history of prior embryo transfer, physician, and embryologist

The overall biochemical pregnancy, clinical pregnancy, and pregnancy loss rates were 80.3%, 70.5%, and 24.5%, respectively. These rates for each quartile are shown in Fig. 2. There were no differences in the probability of biochemical pregnancy, clinical pregnancy, or pregnancy loss between any quartile groups in the fully adjusted models (Fig. 3).

### Sensitivity analyses

When data was analyzed using only first FETs, compared to the shortest embryo transfer time quartile (1st), there was a significantly decreased probability in live birth in the 3rd quartile (OR 0.63, 95%CI 0.41–0.94, *p* = 0.025), but there were no significant differences in the 2nd or 4th quartile groups (*p* = 0.213, *p* = 0.080). One hundred and five (4.6%), 102 (4.5%), and 104 (4.6%) cycles used a stylet, had the transfer catheter handed back to the embryologist, or were described as a difficult embryo transfer, respectively. When patients with these transfer characteristics inherently associated with a longer ET time were excluded, the results were the same as the primary model with the longest (4th) quartile showing significantly decreased probability of live birth (aOR 0.75 (95% CI 0.56–0.99), *p* = 0.049).

Returned or reloaded embryos were additional variables that may have contributed to a longer ET time. There were 12 embryos returned to the embryologist and 32 embryos that needed to be reloaded by the embryologist. All 12 returned embryos were in the longest time quartile. Similarly, most of the embryo reloads occurred in the longest quartile (28/32 embryos), while 2 occurred in the shortest quartile and 1 in each of the middle quartiles. After all returns and reloads were excluded from the analysis, there was no longer a difference in the probability of live birth. Although the sample size is notably small, the LBRs of embryos returned or reloaded were lower, but not statistically different from those without a return or reload (54.6% v. 59.8%, *p* = 0.671; 43.8% v. 60.0%, *p* = 0.062, respectively).

When the analysis was repeated restricting to only good quality embryos (AA or AB) determined by the SART embryo grades, there was still a significant decreased probability of live birth in the 4th quartile (OR 0.54, 95% CI 0.32–0.92, *p* = 0.022), but not in other quartiles when quartile 1 was the reference group. When restricting to only fair or poor quality embryos, no difference in live birth was seen in any quartiles when quartile 1 was the reference group.

As the last (4th) quartile (*n* = 562) had the widest time range, this group was examined more closely. Unsurprisingly, this group was noted to have a highly skewed distribution with a median time of 80 s (IQR 62–127 s). In order to look at this quartile with more granularity, this group was divided further into four subgroups: 287 ETs (transfer time between 55 and 80 s), 125 ETs (80–120 s), 106 ETs (121–300 s), and 44 ETs (301–1028 s). Seventy-three percent of the ETs in the last (4th) quartile were 2 min or less (412 of 562 ETs), while only 7% (44 of 562 ETs) were longer than 5 min. For the shortest time interval group of the 4th quartile (*n* = 287, 55–80 s), the LBR, CPR, and pregnancy loss rates were 55.8%, 69.7%, and 28.6%, respectively, whereas for the longest time interval group of the 4th quartile (*n* = 44, 301–1028 s), the LBR, CPR, and pregnancy rates were 47.7%, 61.4%, and 34.4%, respectively. Although the subgroup with the longest transfer times in this 4th quartile had worse pregnancy outcomes, these differences were not statistically significant (*p* = 0.482; *p* = 0.555; *p* = 0.368; for rates of live birth, clinical pregnancy, and pregnancy loss, respectively).

## Discussion

When evaluating a population of patients undergoing autologous, single, euploid, frozen embryo transfers, our findings demonstrated that transfers with the longest duration were associated with a significantly decreased probability of live birth after adjusting for confounders. However, there were no significant differences observed in the probability of biochemical pregnancy, clinical pregnancy, or pregnancy loss. These results suggest that while prolonged transfer times may impact live birth outcomes, the effects on other pregnancy outcomes remain less clear and warrant further investigation.

The lack of significant associations between the probability of biochemical pregnancy, clinical pregnancy, or pregnancy loss with longer ET times may suggest that while transfer time influences long-term pregnancy outcomes such as live birth, there may not be detectable differences in the initial stages of pregnancy once the embryo has implanted. However, these longer transfer times may expose embryos to suboptimal conditions, such as temperature and pH fluctuations, uterine contractions, or mechanical stress, which could impact their long-term viability and ability to initially implant [15–20]. We furthermore demonstrated that there may be a certain time “threshold” where significant exposure to the extra-uterine and extra-embryologic culture environment must be met prior to seeing adverse effects on pregnancy outcomes, as there were no differences in the probability of live birth in the 2nd or 3rd time quartile cohorts when compared to the 1st (shortest) time quartiles. In stratified analyses focused on overall SART embryo morphology rankings, we saw the same results as the primary analysis when restricting to good quality embryos, but not in fair or poor quality embryos. This suggests that embryo quality likely has a larger impact on final pregnancy outcome when the quality is low compared to the total duration of the transfer.

Similar to the investigation by Lee et al., our study included only frozen blastocyst transfers and controlled for similar confounders such as maternal age and fertility diagnosis. However, our findings did support a significant association between the longest ET times and a lower probability of live birth [13]. One reason for this discrepancy may be due to the fact that this study’s sample size is significantly larger (2267 FET cycles compared to only 465 FETs in Lee et al.), improving the likelihood that significant associations can be detected. Furthermore, Lee et al. included DETs, donor gametes, non-PGT-A tested embryos, and slow-freezing cryopreservation, an older embryology technology that has been proven to have lower thaw survival and possibly affect embryo viability compared to vitrification utilized in this study, the standard embryology practice today [13]. Additionally, by inclusion of only euploid embryos, we were able to account for embryo ploidy as a significant predictive factor on transfer success and better isolate the impact of transfer time.

Our study poses several strengths. The large sample size and inclusion of an array of standardized transfer protocols increases the generalizability of our results. In addition, the use of standardized embryology and grading protocols and study time frame of less than 1 year prohibits significant deviation in culture, grading, or practice techniques that could bias results. Furthermore, all transfers were single frozen blastocyst transfers at a large academic-affiliated clinic performed by 18 different physicians. This study design accounts for variations in clinical practice and technique among a large group of physicians, while also minimizing institutional variability as all transfers followed the same general protocols enhancing the internal consistency of the data. The use of vitrification reflects current standard practice, making these results more generalizable compared to older studies utilizing outdated protocols and technology. Another significant strength of this study is the inclusion of a large number of potential confounders including the physician and embryologist performing the transfer as well as individual patient, cycle, embryo, and transfer characteristics. Finally, the addition of sensitivity and restriction analyses to better isolate the effect of transfer time itself further support the association between longer ET time and decreased probability of live birth.

However, this study also has its limitations, the largest being its retrospective nature. This limitation is likely dampened by the large sample size and robust data analysis that included both multivariate logistic regression and sensitivity analyses to control for multiple confounders and isolate the effect of ET time. Since this study was performed at a single institution, the application of these data to other centers may be limited; however, the inclusion of multiple transfer protocols and large number of physicians with different training backgrounds helps extend applicability. Furthermore, these results cannot be generalized to fresh transfers or cleavage stage transfers, which are still utilized at some institutions.

The existing data on ET time is not sufficient and certainly not reflective of current practice. These results support that the longest transfer times are associated with significantly decreased probability of live birth. Therefore, optimizing ET times to potentially improve LBRs may be warranted. However, because other outcomes like biochemical pregnancy rate, clinical pregnancy rate, and pregnancy loss rate did not show significant differences, immediate changes in clinical practice could be premature. Further research is needed to confirm these trends and to determine if specific ET duration thresholds exist that could maximize success rates. Future research should also focus on elucidating the relationship between ET time and neonatal and obstetrical outcomes. Until then, clinicians might benefit from being mindful of ET time, but should prioritize a patient-centered approach that considers individual procedural factors.

In conclusion, this study found a significant association between prolonged ET times and decreased probability of live birth, suggesting that time-sensitive factors during the ET procedure could impact the likelihood of successful implantation and subsequent development. Future research should focus on identifying the mechanisms by which extended transfer time might affect LBR, as well as exploring potential interventions to optimize ET efficiency. Future studies should also investigate whether specific patient populations are more sensitive to transfer time variations and assess whether these findings apply broadly across different IVF clinic settings. The ultimate goal of ART is a healthy live birth; therefore, optimizing each step in the process has the ability to increase success rates for patients undergoing treatment. If extended ET times reduce the likelihood of live birth, understanding why this happens and how to minimize delays during the transfer process warrants further investigation. These results suggest that IVF transfer protocols can be refined offering patients a higher chance of success, which is especially valuable given the emotional, physical, and financial challenges of infertility treatment.

## Supplementary Information

Below is the link to the electronic supplementary material.

Supplementary file1 (DOCX 228 kb)

Supplementary file2 (DOCX 16 kb)

## Data Availability

This manuscript’s data will not be deposited.
