# Supplementary material for: Every second counts: the association of embryo transfer duration with live birth following 2267 single, euploid, frozen embryo transfer
Source: J Assist Reprod Genet. 2025 Jun 5;42(8):2629–39. doi: 10.1007/s10815-025-03520-7 (PMC12423367; doi:10.1007/s10815-025-03520-7)
Supplement: Supplementary file 1 — Supplementary file1 (DOCX 228 kb) [file 10815_2025_3520_MOESM1_ESM.docx]

**Supplemental Figure 1: Measures of Central Tendency for Embryo Transfer Times in each Quartile**

| 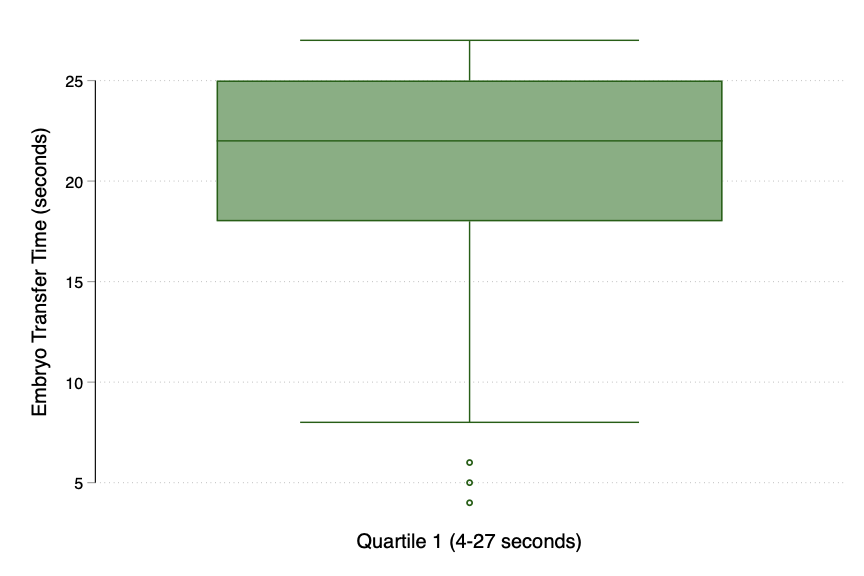 | 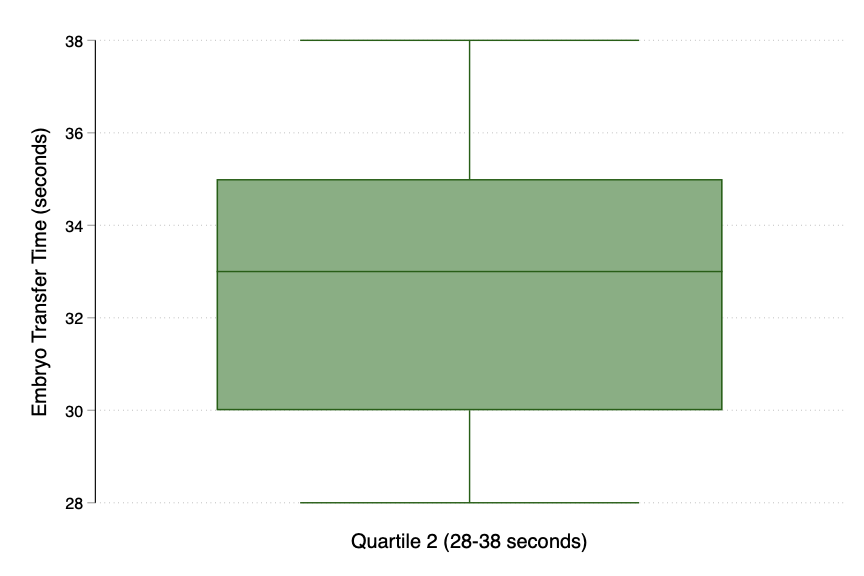 |
| --- | --- |
| 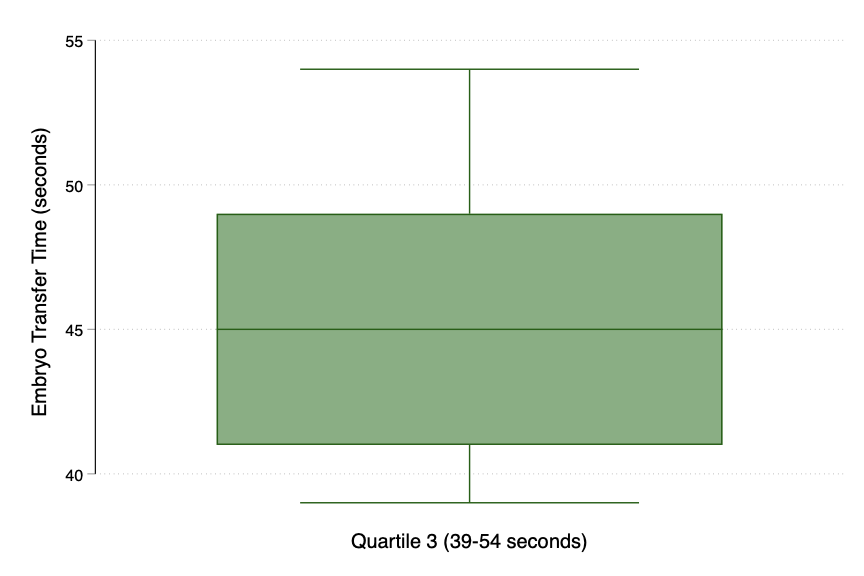 | 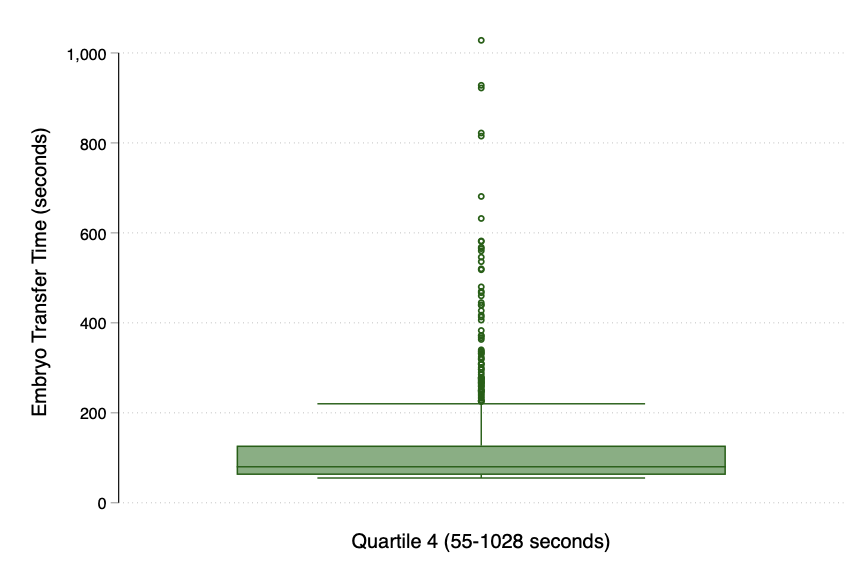 |

Box plots showing the median, inter-quartile range (IQR), minimum and maximum values of the embryo transfer times for each quartile.
