# Supplementary material for: Every second counts: the association of embryo transfer duration with live birth following 2267 single, euploid, frozen embryo transfer
Source: J Assist Reprod Genet. 2025 Jun 5;42(8):2629–39. doi: 10.1007/s10815-025-03520-7 (PMC12423367; doi:10.1007/s10815-025-03520-7)
Supplement: Supplementary file 2 — Supplementary file2 (DOCX 16 kb) [file 10815_2025_3520_MOESM2_ESM.docx]

**Supplemental Table 1A: Distribution of Embryo Transfer Times by Physician**

|  | Total | 1st Quartile (4-27 seconds) | 2nd Quartile (28-38 seconds) | 3rd Quartile (39-54 seconds) | 4th Quartile (55-1028 seconds) |
| --- | --- | --- | --- | --- | --- |
|  | **N=2,267** | **N=593** | **N=577** | **N=535** | **N=562** |
| Physician 1 | 262 (11.56%) | 79 (13.32%) | 70 (12.13%) | 52 (9.72%) | 61 (10.85%) |
| Physician 2 | 129 (5.69%) | 37 (6.24%) | 41 (7.11%) | 31 (5.79%) | 20 (3.56%) |
| Physician 3 | 66 (2.91%) | (1.52%) | 20 (3.47%) | 16 (2.99%) | 21 (3.74%) |
| Physician 4 | 148 (6.53%) | 9 (1.52%) | 32 (5.55%) | 49 (9.16%) | 58 (10.32%) |
| Physician 5 | 232 (10.23%) | 9 (1.52%) | 39 (6.76%) | 96 (17.94%) | 88 (15.66%) |
| Physician 6 | 144 (6.35%) | 69 (11.64%) | 25 (4.33%) | 18 (3.36%) | 32 (5.69%) |
| Physician 7 | 17 (7.90%) | 111 (18.72%) | 29 (5.03%) | 17 (3.18%) | 22 (3.91%) |
| Physician 8 | 54 (2.38%) | 10 (1.69%) | 20 (3.47%) | 10(1.87%) | 14 (2.49%) |
| Physician 9 | 126 (5.56%) | 8 (1.35%) | 50 (8.67%) | 50 (9.35%) | 18 (3.20%) |
| Physician 10 | 179 (7.90%) | 79 (13.32%) | 40 (6.93%) | 26 (4.86%) | 34 (6.05%) |
| Physician 11 | 13 (0.57%) | 1 (0.17%) | 3 (0.52%) | 5 (0.93%) | 4 (0.71%) |
| Physician 12 | 82 (3.62%) | 10 (1.69%) | 24 (4.16%) | 18 (3.36%) | 30 (5.34%) |
| Physician 13 | 176 (7.76%) | 69 (11.64%) | 54 (9.36%) | 28 (5.23%) | 25 (4.45%) |
| Physician 14 | 66 (2.91%) | 4 (0.67%) | 16 (2.77%) | 22 (4.11%) | 24 (4.27%) |
| Physician 15 | 101 (4.46%) | 13 (2.19%) | 32 (5.55%) | 32 (5.98%) | 24 (4.27%) |
| Physician 16 | 56 (2.47%) | 2 (0.34%) | 10 (1.73%) | 19 (3.55%) | 25 (4.45%) |
| Physician 17 | 99 (4.37%) | 43 (7.25%) | 27 (4.68%) | 14 (2.62%) | 15 (2.67%) |
| Physician 18 | 155 (6.84%) | 31 (5.23%) | 45 (7.80%) | 32 (5.98%) | 47 (8.36%) |

**Supplemental Table 1B: Distribution of Embryo Transfer Times by Embryologist**

|  | Total | 1st Quartile (4-27 seconds) | 2nd Quartile (28-38 seconds) | 3rd Quartile (39-54 seconds) | 4th Quartile (55-1028 seconds) |
| --- | --- | --- | --- | --- | --- |
|  | **N=2,267** | **N=593** | **N=577** | **N=535** | **N=562** |
| Embryologist 1 | 103 (4.54%) | 28 (4.72%) | 23 (3.99%) | 29 (5.42%) | 23 (4.09%) |
| Embryologist 2 | 61 (2.69%) | 12 (2.02%) | 17 (2.95%) | 12 (2.24%) | 20 (3.56%) |
| Embryologist 3 | 321 (14.16%) | 94 (15.85%) | 77 (13.34%) | 61 (11.40%) | 89 (15.84%) |
| Embryologist 4 | 182 (8.03%) | 50 (8.43%) | 48 (8.32%) | 33 (6.17%) | 51 (9.07%) |
| Embryologist 5 | 210 (9.26%) | 59 (9.95%) | 53 (9.19%) | 52 (9.72%) | 46 (8.19%) |
| Embryologist 6 | 108 (4.76%) | 34 (5.73%) | 24 (4.16%) | 24 (4.49%) | 26 (4.63%) |
| Embryologist 7 | 135 (5.96%) | 34 (5.73%) | 38 (6.59%) | 31 (5.79%) | 32 (5.69%) |
| Embryologist 8 | 300 (13.23%) | 77 (12.98%) | 69 (11.96%) | 72 (13.46%) | 82 (14.59%) |
| Embryologist 9 | 30 (1.32%) | 7 (1.18%) | 6 (1.04%) | 11 (2.06%) | 6 (1.07%) |
| Embryologist 10 | 152 (6.70%) | 40 (6.75%) | 43 (7.45%) | 34 (6.36%) | 35 (6.23%) |
| Embryologist 11 | 266 (11.73%) | 64 (10.79%) | 78 (13.52%) | 68 (12.71%) | 56 (9.96%) |
| Embryologist 12 | 124 (5.47%) | 30 (5.06%) | 32 (5.55%) | 34 (6.36%) | 28 (4.98%) |
| Embryologist 13 | 275 (12.13%) | 64 (10.79%) | 69 (11.96%) | 74 (13.83%) | 68 (12.10%) |

Data are presented as n (%).
